# Rooting for survival: OsCDPK5 and OsCDPK13 are crucial for rice acclimation to low-oxygen conditions

**DOI:** 10.1093/plphys/kiae359

**Published:** 2024-06-27

**Authors:** Alicja B Kunkowska

**Affiliations:** Assistant Features Editor, Plant Physiology, American Society of Plant Biologists; PlantLab, Institute of Life Sciences, Sant’Anna School of Advanced Studies, 56010 Pisa, Italy

The ability of rice to survive prolonged submergence is a vital component of agricultural progress in regions susceptible to flooding. Rice employs several coping strategies, including molecular reprogramming and physiological changes such as the formation of inducible aerenchyma and the development of adventitious roots from non-root tissues like stem nodes (Voesenek and Bailey-Serres 2015). These root-remodeling processes are facilitated by programmed cell death (PCD). In aerenchyma, PCD is followed by the lysis and disintegration of root cortical cells, creating interconnected air-filled voids that allow for internal oxygen diffusion (Evans 2004). In the development of adventitious roots, cell death in the epidermal cells covering the root primordia enables and promotes the emergence of these roots (Sauter 2013).

The PCD is triggered by the accumulation of reactive oxygen species (ROS) induced by the enhanced function of the NADPH oxidase RESPIRATORY BURST OXIDASE HOMOLOG (RBOH) (Yamauchi et al. 2017). RBOHs are membrane-bound enzymes involved in cell growth and plant development (Chaouch et al. 2012). They produce O_2_^•−^, which dismutates into H_2_O_2_, serving as a messenger in ROS-mediated biotic and abiotic stress responses, including adventitious root outgrowth (Mittler et al. 2022). RBOH-mediated ROS production is activated by CALCIUM-DEPENDENT PROTEIN KINASEs (CDPKs/CPKs) from subgroup I (Adachi and Yoshioka 2015). This subgroup consists of 11 proteins, and it has been demonstrated that 2 of them, CDPK5 and CDPK13, coexpress with rice RBOHH (OsRBOHH) and induce strong ROS production in *N. benthamiana* leaves (Yamauchi et al. 2017). However, much detail about the mechanism of action for these 2 kinases during low-oxygen stress remains to be explored. In this issue of *Plant Physiology*, **Jingxia Li and colleagues (Li et al. 2024)** present crucial evidence for CDPKs’ involvement in rice acclimation to low-oxygen conditions.

To identify phosphorylation sites in OsRBOHH, the authors first analyzed potential CDPK phosphorylation motifs in the literature. Previous studies indicated that ROS production is induced by the phosphorylation of potato StRBOHB by StCDPK4 and StCDPK5 at the Ser82 and Ser97 residues (Kobayashi et al. 2007). Similarly, the authors found orthologous motifs in OsRBOHH, Ser92, and Ser107. To thoroughly investigate the potential phosphorylation of OsRBOHH at these residues by OsCDPK5 and/or OsCDPK13, Li and colleagues (Li et al. 2024) generated different variants of each protein. They created 3 distinct variants of the OsRBOHH to assess their susceptibility to phosphorylation: substituting serine with alanine at position Ser92, Ser107, or both. They also utilized constitutively active forms of OsCDPKs (OsCDPK5-VK and OsCDPK13-VK; Harper et al. 1994) and forms lacking kinase activity (Yamauchi et al. 2017). Additionally, the authors generated antibodies against the phosphorylated Ser92 (pSer92) and Ser107 (pSer107) sites in OsRBOHH. Equipped with these tools, Li and colleagues (Li et al. 2024) conducted several experiments to explore the details of OsRBOHH phosphorylation and its biological consequences.

Initial results showed that both Ser92 and Ser107 are phosphorylated by either OsCDPK5 or OsCDPK13, in parallel with increased ROS production. Furthermore, Ala mutations of one or the other Ser reduced phosphorylation and suppressed ROS production. These findings suggest that OsCDPK5 and OsCDPK13 function redundantly and that phosphorylation of both serine residues in OsRBOHH is required for full ROS production. This conclusion was further supported by an in vitro kinase assay in the presence of a Ca^2+^ chelator, which, by repressing CDPKs activity, almost completely blocked the phosphorylation of OsRBOHH.

**Figure 1.**
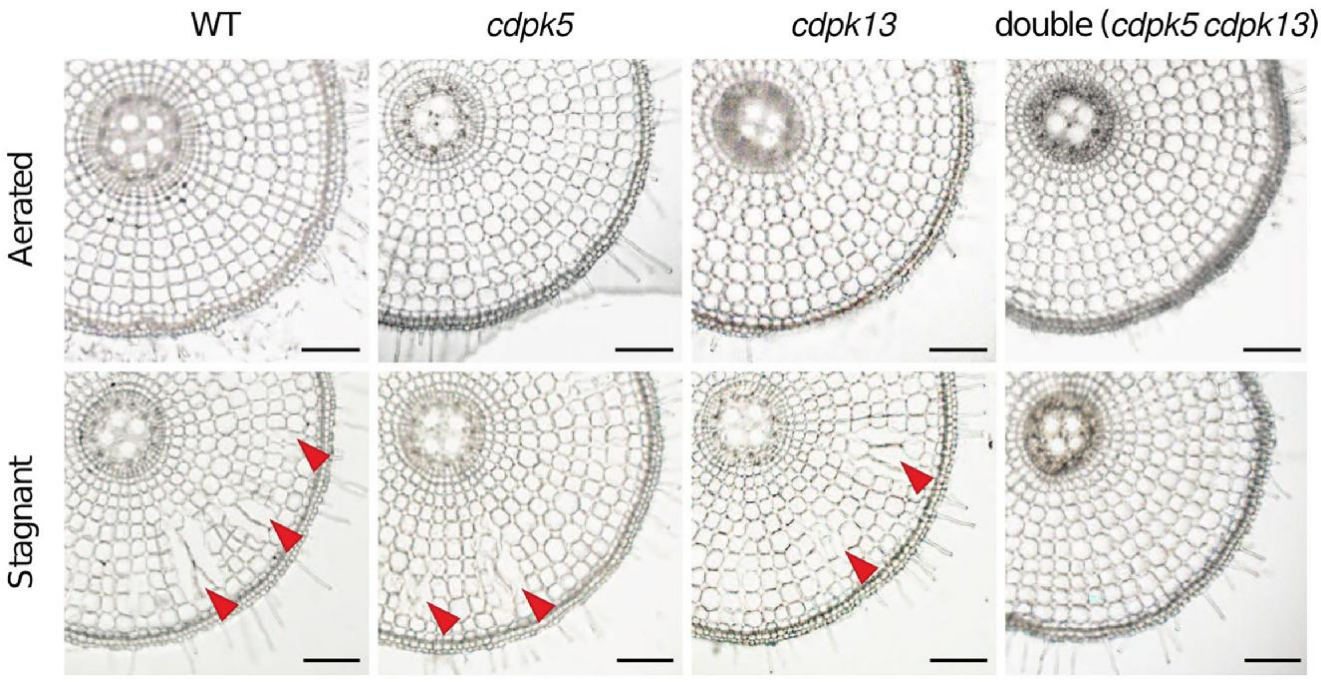
Cross-sections from the tips of adventitious roots were analyzed for the wild type, *cdpk5*, *cdpk13*, and *cdpk5 cdpk13* mutants under both aerated and stagnant (low oxygen) conditions. Red arrows highlight the presence of aerenchyma. Adapted from (Li et al. 2024), Figure 4.

To investigate the role of OsCDPK5 and OsCDPK13 in rice acclimation to low-oxygen conditions, the authors generated single knockout lines (*cdpk5* and *cdpk13*) and a double knockout line (*cdpk5 cdpk13*) in rice, using the CRISPR/Cas9 system. Interestingly, both single knockout lines exhibited reduced levels of aerenchyma formation, while the double knockout line showed an almost complete suppression of aerenchyma formation (Fig. 1). These morphological alterations are likely caused by changes in ROS homeostasis. Under the low-oxygen conditions, while H_2_O_2_ levels increased in the wild-type rice, they were significantly reduced in the *cdpk5 cdpk13* mutant. Additionally, the nitroblue tetrazolium staining detected less O_2_^•−^ in the *cdpk5 cdpk13* mutant under low-oxygen conditions than in wild type. Moreover, the microscope pictures showed a lower number of collapsed cells in the double mutant, indicating decreased aerenchyma formation.

The *cdpk5 cdpk13* mutant had fewer adventitious roots than the wild type even under aerated conditions, with the difference becoming more pronounced under low-oxygen conditions. Remarkably, in all morphological analyses, the single *cdpk5* and *cdpk13* mutants resembled the wild type, further supporting the conclusion that both kinases act redundantly.

Together, the research conducted by Li and colleagues (Li et al. 2024) unveiled a precise mechanism of ROS and Ca^2+^ signaling in rice acclimation to low-oxygen stress. Low-oxygen conditions strongly induce the expression of *OsRBOHH*. Active forms of OsCDPK5 and OsCDPK13 phosphorylate OsRBOHH at the Ser92 and Ser107 residues, stimulating an ROS burst that triggers inducible aerenchyma formation and contributes to the development of adventitious roots. This process enhances oxygen diffusion, benefiting rice acclimation to low-oxygen environments. Future studies may focus on assessing whether other OsRBOHs are involved in these processes.
